# Effectiveness of Ozone Therapy in Botulinum Toxin–Induced Ptosis: Two Case Reports

**DOI:** 10.1111/jocd.70729

**Published:** 2026-02-06

**Authors:** Selda Yıldırım Gençtürk, Muhammed Burak Yücel, Çağlar Gençtürk

**Affiliations:** ^1^ Private Dr. Selda Yıldırım Gençtürk Dermatology Clinic İstanbul Turkey; ^2^ Dermatology Clinic, Ergani State Hospital Diyarbakır Turkey

## 1 Introduction

Ozone has been used empirically and as an alternative medical modality for more than a century, and recent technological advancements have provided new insights into its therapeutic potential [1]. Ozone therapy, a noninvasive, low‐cost treatment with a favorable side‐effect profile, has been increasingly utilized in various dermatologic and esthetic indications [2]. The existing literature reports the use of oxygen–ozone mixtures in the management of localized adiposity, cellulite, wrinkles, skin laxity, acne, hyperpigmentation, striae, and telangiectasia [3]. Here, we present the clinical outcomes of intradermal ozone therapy in two patients who developed eyelid ptosis following botulinum toxin injection.

## 2 Case Reports

### 2.1 Therapeutic Procedure

For both cases, the ozone gas was generated using a medical‐grade ozone generator (HAS Salutem, Cemil Has Medikal, Izmir, Turkey). The device possesses EC certification in accordance with the Medical Device Directive 93/42/EEC and is manufactured under EN ISO 13485:2016 quality management standards. This system allows for precise photometric measurement of ozone concentration to ensure dosage accuracy. The gas was collected in a siliconized syringe immediately prior to injection to ensure concentration stability, and a 30G needle was used for administration.

### 2.2 Case 1

**FIGURE 1:**
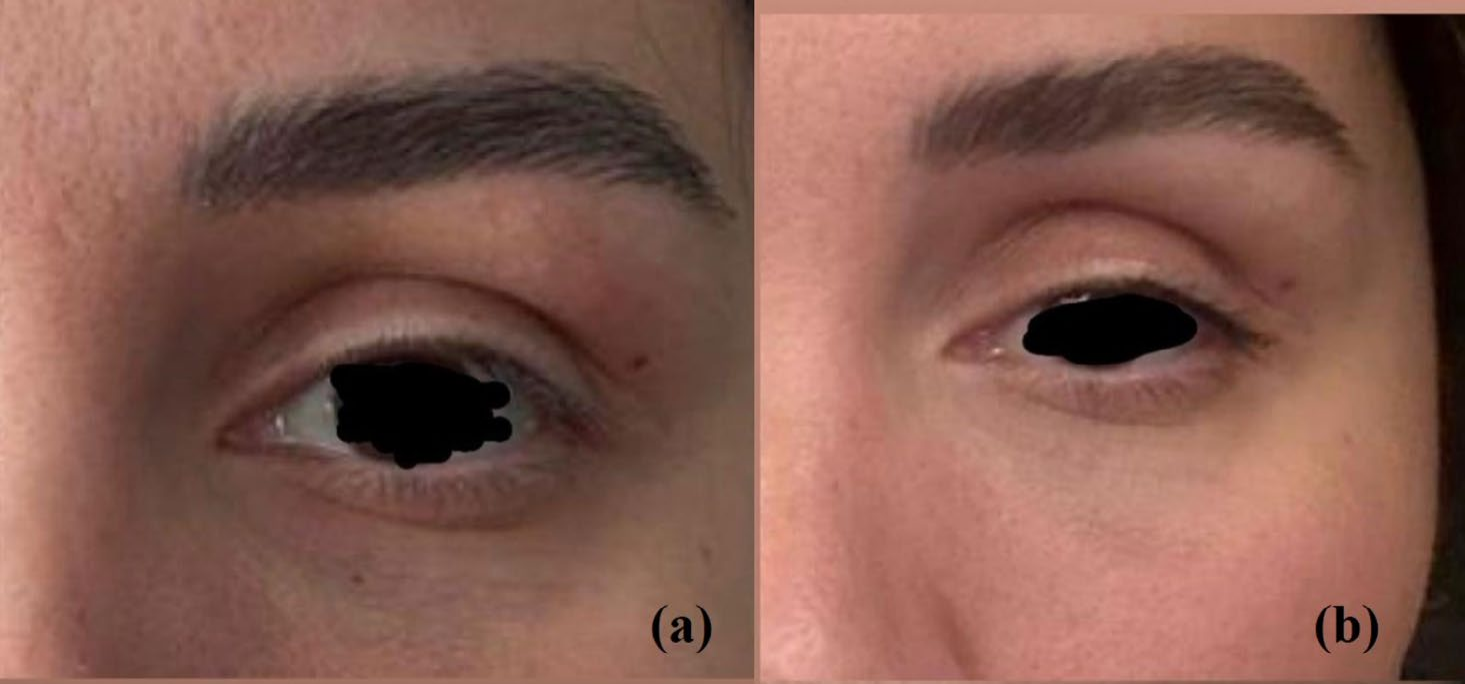
(a, b) Clinical photographs of Case 1. (a) Left upper eyelid ptosis developing approximately 1 week after abobotulinum toxin injection (Day 0 of ozone treatment). (b) Marked improvement in ptosis observed 5 days after the initiation of ozone therapy (Day 5).

A 36‐year‐old woman developed left upper eyelid ptosis approximately 1 week after receiving abobotulinum toxin. A nondermatology practitioner presumed an allergic reaction and initiated systemic corticosteroids; however, the patient showed no clinical improvement and presented to our clinic with persistent symptoms. Dermatologic examination revealed left upper eyelid drooping and mild asymmetry of the periorbital muscles (Figure 1a).

As treatment, a total of 2.5 mL of ozone at a concentration of 3 gamma was administered intradermally and intramuscularly into the corrugator muscle (body and tail), the upper eyelid, and the periorbital region. Each site received approximately 0.1–0.2 mL, delivered in two sessions spaced 2 days apart. No additional therapy was provided.

Five days later, a marked improvement in ptosis was observed (Figure 1b), and no adverse events occurred.

### 2.3 Case 2

**FIGURE 2:**
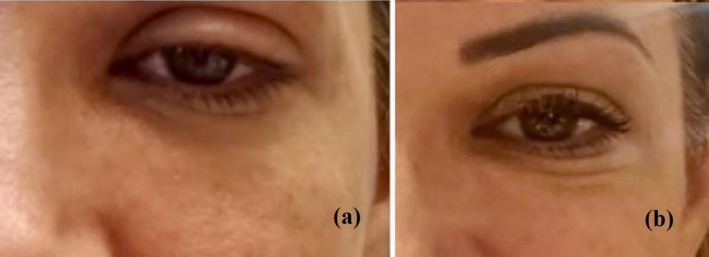
(a, b) Clinical photographs of Case 2. (a) Left upper eyelid ptosis appearing on the second day following abobotulinum toxin injection (Day 0 of ozone treatment). (b) Rapid improvement in ptosis observed on the second day following the first session of ozone therapy (Day 2).

A 48‐year‐old woman presented with left upper eyelid ptosis that developed on the second day following abobotulinum toxin injection. The patient reported taping her eyelid to manage daily activities. Dermatologic examination confirmed left upper eyelid ptosis (Figure 2a).

As treatment, a total of 2.5 mL of ozone at a concentration of 3 gamma was administered intradermally and intramuscularly into the corrugator muscle (body and tail), the upper eyelid, and the periorbital region. Each site received approximately 0.1–0.2 mL, delivered in two sessions spaced 2 days apart. No additional treatment was given.

By the 2nd day after treatment, a marked improvement in ptosis was observed (Figure 2b), and no adverse events were detected.

## 3 Discussion

Ptosis of the upper eyelid may occur after botulinum toxin injections to the glabellar region and its surroundings due to unintended diffusion of the toxin. The toxin can traverse the orbital septum and induce weakness in the levator palpebrae superioris muscle. Upper eyelid ptosis typically appears 7–10 days after injection and may persist for 2–4 weeks or longer. Therapeutic options include topical sympathomimetic agents such as 0.5% apraclonidine or phenylephrine ophthalmic solution, which stimulate Müller's muscle and elevate the eyelid. Muscle activation techniques including targeted exercises, mechanical stimulation, or electrical stimulation may also help shorten the duration of ptosis [4, 5].

Ozone gas (O_3_), a potent oxidizing agent, possesses anti‐inflammatory, antioxidant, and tissue‐reparative properties. The literature indicates that ozone is effective against bacteria, viruses, fungi, and protozoa; that short‐term, low‐ to moderate‐dose exposure stimulates endogenous antioxidant systems; and that such exposure does not induce tissue injury. Ozone can be administered intravenously, intramuscularly, subcutaneously, intradermally, or locally. When applied locally to the skin, it enhances circulation, improves impaired biological functions, and increases cytokines involved in tissue repair, such as IFN‐β and TGF‐β [6, 7]. Recent experimental work further supports these pleiotropic effects of ozone. In a stable ozonized glycerin hydrogel, Russo et al. reported not only broad antimicrobial and antibiofilm activity against multidrug‐resistant Gram‐positive, Gram‐negative, and Candida isolates, but also a significant reduction in TNF‐α production by LPS‐stimulated human peripheral blood mononuclear cells and enhanced migratory/proliferative capacity of dermal fibroblasts and keratinocytes in scratch assays [8]. These findings are consistent with the anti‐inflammatory and tissue‐regenerative properties postulated to underlie the rapid clinical improvement observed in our patients and provide additional biological plausibility for the use of ozone formulations as adjunctive therapies in cutaneous complications.

The rapid clinical improvement observed in our cases (2–5 days) contrasts with the typical natural history of botulinum toxin–induced ptosis, which generally persists for 2–4 weeks. While direct inactivation of the toxin is unlikely given the timing, the rapid response may be attributed to ozone's anti‐inflammatory properties [9]. Localized edema and inflammation can exacerbate the mechanical weight on the eyelid, worsening ptosis. By downregulating pro‐inflammatory cytokines (TNF‐α, IL‐12) and reducing local tissue edema, ozone therapy may alleviate this burden, allowing for faster functional recovery of the levator muscle even if the toxin's paralytic effect has not fully resolved.

In our cases, intradermal and intramuscular ozone therapy administered to the corrugator muscle, upper eyelid, and periorbital region resulted in rapid and marked improvement in ptosis, without adverse effects. These outcomes suggest that ozone therapy may represent a promising adjunctive option in the management of botulinum toxin–related complications. Although direct evidence for ozone in the treatment of blepharoptosis is limited, indirect support exists in the literature. In a randomized, comparative study involving patients with piriformis syndrome, intramuscular ozone injection produced pain and disability improvements within 1–2 months comparable to lidocaine and more rapid than botulinum toxin; at 3–6 months, however, botulinum toxin was superior. These findings imply that ozone may accelerate short‐term clinical improvement through its anti‐inflammatory and analgesic effects. Nevertheless, as the study addressed a musculoskeletal condition rather than ptosis, the evidence remains indirect and primarily supports biological plausibility. Controlled studies specifically evaluating ozone for blepharoptosis are required to establish its efficacy [10].

Several limitations must be acknowledged. First, this report is limited to two cases without a control group. Given that botulinum toxin–induced ptosis is a self‐limiting condition, spontaneous resolution cannot be definitively excluded, although the speed of recovery observed here is atypical for the natural course. Second, the assessment of ptosis was based on clinical examination and patient satisfaction rather than objective measurements such as Margin Reflex Distance 1 (MRD1). Finally, subjective factors such as compensatory frontalis muscle activation or changes in lighting in photographs could influence the perceived degree of improvement.

In conclusion, intradermal and intramuscular ozone therapy represents a promising adjunctive approach in the management of botulinum toxin–induced ptosis. Our observations suggest it may accelerate recovery, possibly through anti‐inflammatory mechanisms and reduction of local edema. However, these findings are preliminary and hypothesis‐generating. Large‐scale, controlled clinical trials with objective outcome measures are necessary to confirm efficacy, safety, and the optimal treatment protocol before it can be recommended as a standard therapy.

## Author Contributions

Muhammed Burak Yücel: conceptualization, data acquisition, clinical management of cases, manuscript drafting, and critical revision. Çağlar Gençtürk: literature review, manuscript editing, clinical interpretation. Selda Yıldırım Gençtürk: supervision, clinical consultation, manuscript revision for important intellectual content. All authors approved the final version of the manuscript and agree to be accountable for all aspects of the work.

## Funding

The authors have nothing to report.

## Disclosure

Both patients provided written informed consent for the off‐label use of ozone therapy and for the publication of their clinical details and photographs. The procedures were conducted in accordance with the ethical principles outlined in the Declaration of Helsinki.

## Ethics Statement

The authors confirm that the ethical policies of the *Journal of Cosmetic Dermatology* have been adhered to. According to institutional guidelines, formal ethics committee approval is not required for case reports involving one or two patients, as long as no identifiable personal data beyond clinical images are used. This study complies with the Declaration of Helsinki principles.

## Consent

Written informed consent was obtained from both patients for the publication of their clinical details and accompanying images.

## Conflicts of Interest

The authors declare no conflicts of interest.

## Data Availability

Data sharing is not applicable, as this article reports individual clinical cases and no datasets were generated or analyzed.
